# Don’t make me blush: msSPX4/MsPHL11-MsWRKY91 regulates anthocyanin synthesis in *Malus spectabilis* under low phosphorus stress

**DOI:** 10.1093/plphys/kiag167

**Published:** 2026-04-29

**Authors:** Catherine P Freed

**Affiliations:** Department of Biochemistry, University of Wisconsin-Madison, Madison, WI 53706, United States; Assistant Features Editor, Plant Physiology, American Society of Plant Biologists

Phosphorus (P) is a vital macronutrient for plant development and reproduction. Plants have evolved a series of complex mechanisms to cope with low phosphorus stress, including the production and accumulation of anthocyanins (Madison et al. 2023). Anthocyanins are a diverse group of pigmented molecules that provide plants with unique red and purple coloration, sometimes referred to as “blushing” (Jezek et al. 2023). Anthocyanins also provide protection against abiotic stresses through scavenging free radicals (Kaur et al. 2023). Understanding the mechanisms of anthocyanin accumulation under P stress is key to enhance crop resilience in times of nutrient scarcity.

P uptake is coordinated by transcription factors, such as PHOSPHATE RESPONSE (PHR/PHL), that activate genes to enhance P accumulation under low P conditions (Rouached et al. 2010; Chien et al. 2018). When P is abundant, SPX proteins bind to PHR/PHL transcription factors, thus inhibiting expression of P-sensitive genes. While the SPX:PHR module responds to low P levels and impacts plant anthocyanin accumulation, the mechanisms interconnecting these two remain unknown.

In a recent study published in *Plant Physiology*, Wei et al. (2026) sought to characterize the link between anthocyanin synthesis and regulation under low phosphorus stress in *Malus spectabilis* (crabapple). Their experiments demonstrated that low P levels increased anthocyanin accumulation in *M. spectabilis* leaves, reaching peak anthocyanin accumulation at 300 μM P. Plants grown on media containing 300 μM P were significantly more stressed as shown by electrolyte leakage as well as increased levels of malondialdehyde, a hallmark of oxidative stress. Their analysis also revealed that the activity of acid phosphatases, proteins that release P from soil, and expression of anthocyanin synthesis genes peaked at 300 μM P. Notably, while plants grown at 0 μM P were more stressed and growth-inhibited than 300 μM P plants, there was no significant difference in anthocyanin levels or expression of anthocyanin synthesis genes between the 2 groups. Taken together, 300 μM P is sufficient for increasing anthocyanin accumulation in *M. spectabilis* and the ideal condition to assess the impact of P starvation on anthocyanin accumulation with a small growth tradeoff.

Wei et al. used RNAseq to query the gene regulatory networks in *M. spectabilis* leaves under replete (1.2 mM P) and 300 μM P (low phosphorus; LP). Their comparative analysis revealed over 6,000 differentially expressed genes enriched under LP conditions with a significant enrichment for differentially expressed genes involved in secondary metabolite and flavonoid synthesis. The authors also identified *MsSPX4* as a potential regulator of anthocyanin accumulation, as it was significantly repressed under LP. Stable apple transgenics and apple peels infiltrated to transiently overexpress *MsSPX4*-silencing and *MsSPX4*-overexpressing (*MsSPX4*-OE) constructs showed that *MsSPX4* silencing increased anthocyanins while *MsSPX4*-OE had decreased anthocyanins. *MsSPX4*-OE apple transgenics also showed a reduced capacity to cope with LP stress, suggesting that *MsSPX4* inhibits anthocyanin accumulation under LP and, as a consequence, negatively impacts plant resilience to LP.

The authors found 3 differentially expressed *MsPHL* genes in their RNAseq dataset, which is notable given SPX proteins inhibit PHR1/PHL1 in other plant species. Bimolecular fluorescence complementation and yeast 2-hybrid assays revealed that MsSPX4 interacted with 2 of the 3 identified MsPHLs: MsPHL11 and MsPHL8. Given *MsPHL8* expression decreased with LP whereas *MsPHL11* was unaffected, the authors hypothesized that the interaction between MsSPX4 and MsPHL11 could impact anthocyanin biosynthesis. Silencing and overexpression of *MsPHL11* showed the inverse phenotype of *MsSPX4* constructs: *MsPHL11* overexpression increased anthocyanins and silencing decreased anthocyanin levels. Overexpression of *MsPHL11* in stable lines decreased leaf stress and increased P accumulation, demonstrating that *MsPHL11* positively regulates anthocyanin accumulation and enhances plant resilience to LP stress.

The authors observed differential expression of 41 WRKY genes, transcription factors that are also involved in P sensing and acquisition (Tang et al. 2023). A subset of these WRKYs had PHR1/PHL1 potential binding sites in the promoter region. Of these, MsPHL11 interacted with the P1BS binding motif of the MsWRKY91 promoter. This interaction suggests that MsPHL11 regulates *MsWRKY91* expression. MsWRKY91 also activated expression of *MsF3′H*, an important gene in the anthocyanin synthesis pathway, by binding to the MsF3′H promoter under low P conditions.

This study identifies a synergistic relationship between MsSPX4/MsPHL11–MsWRKY91 to regulate anthocyanin accumulation under LP conditions in *M. spectabilis* (Fig. 1). MsPHL11 indirectly increases anthocyanin accumulation by inducing MsWKRY9 to positively regulate synthesis of anthocyanin accumulation genes, like *MsF3′H*. This module is in turn negatively regulated by MsSPX4 as MsSPX4 physically inhibits MsPHL11 from binding to MsWRKY91 promoter sequences. Validating this module in other plant and crop species merits further investigation. Future efforts exploring whether overexpressing parts of this module, such as *MsPHL11* or *MsWRKY91*, could improve crop growth under nutrient stress without a negative growth or defense tradeoff would also be insightful.

**Figure 1:**
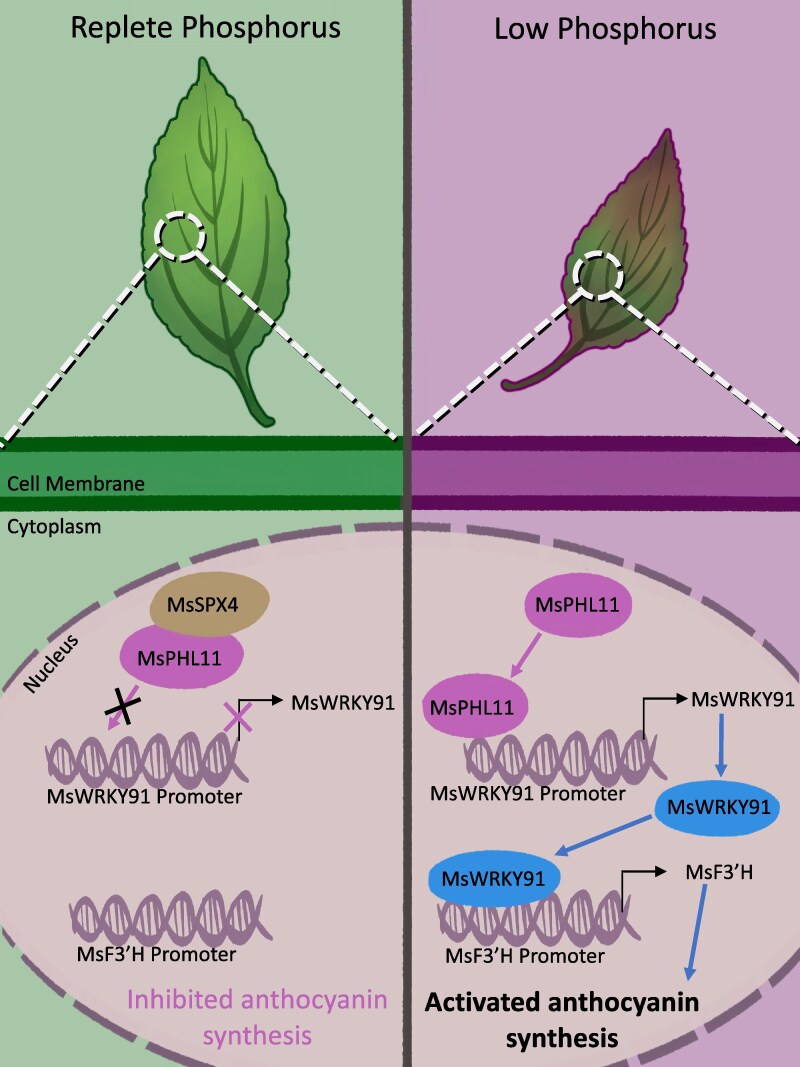
Model depicting a hypothetical MsSPX4/MsPHL11-MsWRKY91 module in anthocyanin synthesis in *M. spectabilis* leaves under replete and low P conditions. When grown on replete P, MsSPX4 prevents MsPHL11 from binding to the MsWRKY91 promoter, suppressing expression of *MsWRKY91* and inhibiting anthocyanin synthesis. Under low P, MsPHL11 promotes expression of *MsWRKY91*, promoting *MsF3'H* expression to activate anthocyanin synthesis. This figure was adapted from Fig. 10 in Wei et al. (2026).

## Data Availability

No new data were generated or analyzed in support of this research.
